# Incidence and survival in non-hereditary amyloidosis in Sweden

**DOI:** 10.1186/1471-2458-12-974

**Published:** 2012-11-13

**Authors:** Kari Hemminki, Xinjun Li, Asta Försti, Jan Sundquist, Kristina Sundquist

**Affiliations:** 1 Division of Molecular Genetic Epidemiology, German Cancer Research Centre (DKFZ), Heidelberg, 69120, Germany; 2 Center for Primary Health Care Research, Lund University, Malmö, Sweden; 3 Stanford Prevention Research Center, Stanford University School of Medicine, California, USA

## Abstract

**Background:**

Amyloidosis is a heterogeneous disease caused by deposition of amyloid fibrils in organs and thereby interfering with physiological functions. Hardly any incidence data are available and most survival data are limited to specialist clinics.

**Methods:**

Amyloidosis patients were identified from the Swedish Hospital Discharge and Outpatients Registers from years 2001 through 2008.

**Results:**

The incidence of non-hereditary amyloidosis in 949 patients was 8.29 per million person-years and the diagnostic age with the highest incidence was over 65 years. Secondary systemic amyloidosis showed an incidence of 1 per million and a female excess and the largest number of subsequent rheumatoid arthritis deaths; the median survival was 4 years. However, as rheumatoid arthritis deaths also occurred in other diagnostic subtypes, the incidence of secondary systemic amyloidosis was likely to be about 2.0 per million. The median survival of patients with organ-limited amyloidosis was 6 years. Most myeloma deaths occurred in patients diagnosed with unspecified or ‘other’ amyloidosis. These subtypes probably accounted for most of immunoglobulin light chain (AL) amyloidosis cases; the median survival time was 3 years.

**Conclusions:**

The present diagnostic categorization cannot single out AL amyloidosis in the Swedish discharge data but, by extrapolation from myeloma cases, an incidence of 3.2 per million could be ascribed to AL amyloidosis. Similarly, based on rheumatoid arthritis death rates, an incidence of 2.0 could be ascribed to secondary systemic amyloidosis.

## Background

Amyloidosis is a heterogeneous group of diseases characterized by fibrillar protein deposits, amyloids, localized in a single organ or systematically in many organs [1-3]. The disease nomenclature is based on the precursors of the amyloid fibrils for which at least 28 different proteins have been identified [1,4,5]. The abbreviations for the amyloids are constructed by starting with an ‘A’ for amyloid and adding a suffix designating the precursor protein, e.g., AL is amyloid derived from immunoglobulin light chain precursor. AL amyloidosis is the respective disease which is the most common systemic amyloidosis [2,4,6]. Other systemic forms include reactive (AA with serum amyloid A as the precursor) and senile (SSA, transthyretin) amyloidosis [4]. AA amyloidosis is a secondary condition in response to chronic inflammation or even cancer; e.g., some 20% of rheumatoid arthritis patients show some amyloid accumulation in the course of their disease [7]. In a re-evaluated Finnish autopsy series on rheumatoid arthritis deaths amyloid was found in 30% of cases and amyloidosis was considered the cause of death in 9.5% of rheumatoid arthritis patients [8,9]. According to causes of death in rheumatoid arthritis patients, 5.8% have been ascribed to amyloidosis but the proportion appears to be declining with time due to a better control of the inflammation [6,10,11]. Alzheimer disease is also a form of amyloidosis but because it is an independent diagnostic entity it will not be considered here. The most common and wide-spread hereditary amyloidosis, familial amyloid polyneuropathy (FAP), is caused by transthyretin mutations [12,13]. However, hereditary amyloidosis is outside the scope of the present study, and will only be referred to in the context of diagnostic specificity for non-hereditary amyloidosis.

The diagnosis is based on the demonstration of amyloid fibrils in a tissue biopsy. In AL amyloidosis, the presence of a monoclonal immunoglobulin, M protein, can usually be demonstrated in serum or urine samples. The symptoms of amyloidosis arise in the critical organs where amyloid accumulates, including the heart, kidney, liver and peripheral nerves [4]. For AL amyloidosis cardiac involvement is the most common life-threatening manifestation. The median survival times depend on the extent of interference with the critical organ functions and these range from some months to some years, based on patient clienteles of expert clinics [2,4]. AL amyloidosis is associated with multiple myeloma and probably also with its precursor disease, monoclonal gammopathy of unknown significance (MGUS); some 10 to 15% of multiple myeloma patients have AL amyloidosis [14,15]. Conversely, some 10% of AL amyloidosis patients have MM at the time of diagnosis [16].

There are limited data on the incidence of amyloidosis probably because of the rarity and heterogeneity of the condition. In 1992 Robert Kyle and coworkers wrote that “…we are unaware of any published studies on the incidence or prevalence of AL…” [17]. In that paper they gave the incidence for AL amyloidosis in a US county overall as 9 per million person-years but the rate was based on 21 patients. This rate has been cited since then because apparently no other incidence data have become available. In the present study we report on the incidence of amyloidosis based on nation-wide hospitalization in Sweden, covering both inpatients and outpatients. Data are given by subtypes between 2001 through 2008.

## Methods

### Source of data

Amyloidosis patients were identified from the Swedish Hospital Discharge Register (2001–2008) or from the Outpatients Register (2001–2008). The Hospital Discharge Register has been operated regionally since 1964 and nationally since 1987. In order to ensure that the reported incidence data included newly hospitalized patients, anyone previously discharged with an amyloidosis diagnosis was excluded. Information from the registers was linked at the individual level via the national 10-digit civic registration number assigned to each person in Sweden for his or her lifetime. In the linked dataset, civic registration numbers were replaced by serial numbers to ensure the anonymity of each individual.

### Outcome variables

The 10th revision of the International Classification of Diseases (ICD-10) was used to identify all first hospital admissions for the outcome variables amyloidosis E85: secondary systemic amyloidosis, E85.3; organ-limited amyloidosis, E85.4; other amyloidosis, E85.8; and amyloidosis, unspecified, E85.9. However, as amyloidosis diagnostics is time consuming, the initial discharge may be changed. We used therefore the discharge diagnosis of the last hospitalization. Diagnostic details are not included among the discharge data.

### Individual variables

Sex: Males and females were included in the study. Geographic region of residence in 1990 was divided into (1) large cities (cities with a population of more than 200,000, i.e., Stockholm, Gothenburg, and Malmö); (2) Southern Sweden; and (3) Northern Sweden. These data were used in the adjustments as detailed below.

### Statistical analysis

Person-years were calculated from the start of follow-up on 1 January 2001, until hospitalization for amyloidosis, death, emigration, or the closing date (31 December 2008). Age-, gender-, region of residence-, immigration status-, and subtypes of diagnosis-specific incidence rates were calculated for the whole follow-up period. Relative weights used to calculate the incidence rates were based on the 2000 European standard population. We used SAS version 9.2 for the statistical analyses.

### Ethical considerations

This study was approved by the Ethics Committee of the Lund University, Sweden.

## Results

Case numbers and incidence of hospitalized amyloidosis patients are shown in Table 1. For years 2001 to 2008, a total of 949 patients were identified, giving an incidence of 8.29 per million person-years. Unspecified amyloidosis was the largest disease category with 535 patients (incidence 4.69/million), follow by secondary systemic amyloidosis (136 patients, 1.18/million). Overall, men had a somewhat higher incidence than women (1.3-fold for unspecified amyloidosis), with the exception of secondary systemic amyloidosis for which the female incidence was 1.9 times the male rate.

**Table 1:** Numbers and incidence rates (per million person- years) for hospitalized amyloidosis patients, 2001-2008

	**Men**				**Women**				**All**			
**Subtype (ICD-10 code)**	**No.**	**IR**	**95% CI**		**No.**	**IR**	**95% CI**		**No.**	**IR**	**95% CI**	
Secondary systemic amyloidosis (E85.3)	50	0.81	0.58	1.03	86	1.57	1.23	1.90	136	1.18	0.98	1.37
Organ-limited amyloidosis (E85.4)	54	0.96	0.70	1.21	57	1.05	0.78	1.32	111	1.00	0.82	1.19
Other amyloidosis (E85.8)	95	1.57	1.26	1.89	72	1.27	0.97	1.56	167	1.42	1.21	1.64
Amyloidosis, unspecified (E85.9)	301	5.25	4.66	5.84	234	4.10	3.58	4.63	535	4.69	4.29	5.09
All	500	8.59	7.84	9.34	449	7.98	7.25	8.72	949	8.29	7.76	8.82

**Figure 1:**
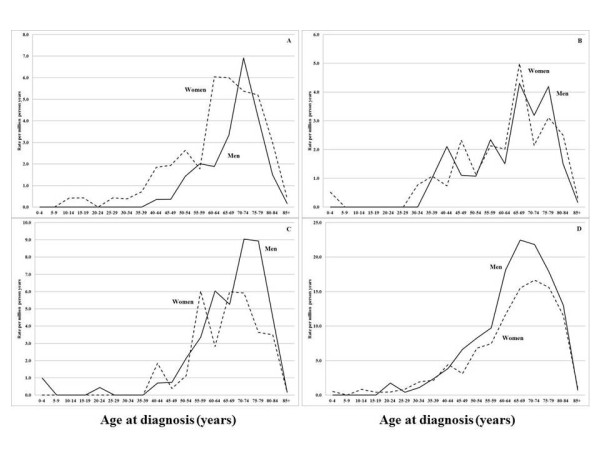
**Age-specific incidence rate (per million person-years) of hospitalized patients with amyloidosis by subtypes, 2001–2008: Figure **1**A secondary systemic amyloidosis (E85.3); Figure **1**B organ-limited amyloidosis (E85.4); Figure **1**C other amyloidosis (E85.8); Figure **1**D amyloidosis, unspecified (E85.9).**

The age-specific rate of hospitalization for amyloidosis is shown in Figure 1. Note that the scales of the y-axes vary. Secondary systemic amyloidosis showed a sharp male peak at age 70–74 years and a broader female peak with younger cases (A). Organ-limited amyloidosis reached a broad male maximum at age 65–79 years and the female maximum somewhat earlier; both male and female curves showed evidence on two main components (B). ‘Other’ amyloidosis types followed the same pattern (C) but for unspecified amyloidosis the male maximum at 65–69 years occurred earlier than the female maximum at 70–74 years (D).

Regional differences in the incidence of amyloidosis were analyzed by residence as of year 1990 (data not shown). The only significant differences (i.e., 95%CI did not overlap with ‘All’, IR 3.84, 95%CI 3.55-4.14) were found for unspecified amyloidosis for the provinces of Uppsala (n= 36, IR 6.93, 95%CI 4.67-9.20) and the neighboring province to the west, Västmanland (n= 38, IR 6.67, 95%CI 4.55-8.78). Importantly, no excess cases were found in the two northernmost provinces which are the endemic area of FAP [13].

Causes of death were recorded for 1143 amyloidosis patients hospitalized between years 1997 through 2008, the longest period when subtypes were recorded (Table 2). A total of 234 (21%) underlying causes of death were due to amyloidosis and 328 (29%) were due to other causes. Additionally, amyloidosis was given as a contributing cause of death for 17 patients. Among other underlying causes, deaths due to cancer were the most common cause and accounted for 16/40 cases in ‘other’ amyloidosis and 68/182 for unspecified amyloidosis. Diseases of the circulatory system predominated for secondary systemic and organ-limited amyloidosis. Myeloma would be pathognomonic of AL amyloidosis and it accounted for 5 (secondary systemic amyloidosis), 0 (organ-limited amyloidosis), 12 (other amyloidosis) and 24 (unspecified amyloidosis) deaths among these four subtypes, respectively. Incident myelomas were recorded for 7, 3, 13 and 21 patients, respectively. Thus, assuming that 10% of AL amyloidosis patients have myeloma at diagnosis [16], the extrapolated AL amyloidosis case numbers were 70 (37% of secondary systemic amyloidosis), 30 (22% of organ-limited amyloidosis), 130 (80% of other amyloidosis) and 210 (32% of unspecified amyloidosis), adding up to 440 cases, which was 38% of all 1143 patients. Rheumatoid arthritis would be indicative of secondary systemic amyloidosis; 14/85 (17%) deaths in secondary systemic amyloidosis were due to rheumatoid arthritis, which was far above unspecified amyloidosis with 10/182 (5%) rheumatoid arthritis deaths.

**Table 2:** Causes of death in hospitalized amyloidosis patients 1997-2008

**Subtype of amyloidosis**	**No. of hospitalized case**	**First cause of death due to amyloidosis**	**Second cause of death due to amyloidosis**	**Other causes of death**
Secondary systemic amyloidosis (E85.3)	190	18	6	85
Organ-limited amyloidosis (E85.4)	137	27	3	21
Other amyloidosis (E85.8)	164	44	4	40
Amyloidosis, unspecified (E85.9)	652	145	4	182
All	1143	234	17	328

Other causes of death in E85.3 patients: 3 certain infections, 16 cancers, 25 diseases of the circulatory system, 3 diseases of the respiratory system, 2 diseases of the digestive system, 1 diseases of the skin and subcutaneous tissue, 17 disease of the musculoskeletal system and connective tissue, 10 disease of the genitourinary system, 5 symptoms and signs and 3 others.

Other causes of death in E85.4 patients: 5 cancers, 1 psychiatry disorder, 11 diseases of the circulatory system, 1 diseases of the digestive system, 1 diseases of the musculoskeletal system and connective tissue and 2 diseases of the genitourinary system.

Other causes of death in E85.8 patients: 2 certain infections, 16 cancers, 1 diseases of the blood and blood-forming organs, 11 diseases of the circulatory system, 1 diseases of the respiratory system, 2 diseases of the digestive system, 2 disease of the musculoskeletal system and connective tissue, 4 disease of the genitourinary system and 1 other.

Other causes of death in E85.9 patients: 3 certain infections, 68 cancers, 1 diseases of blood and blood-forming organs, 6 endocrine, nutritional and metabolic diseases, 1 psychiatry disorder, 2 diseases of nervous system, 45 diseases of the circulatory system, 8 diseases of the respiratory system, 20 diseases of the digestive system, 10 disease of the musculoskeletal system and connective tissue, 6 disease of the genitourinary system, 5 symptoms and signs and 7 other.

For the estimation of the overall hospitalization rate for amyloidosis, we compared previous hospitalizations of the 197 patients whose underlying cause of death was amyloidosis in years 2005 through 2008. Of these, 151 were hospitalized any time from 1964 onwards for amyloidosis as the main or secondary diagnosis. Thus the hospitalization rate was at least 77% for this group of patients. However, the true rate is likely to be higher because the Hospital Discharge Registry reached nation-wide coverage not earlier than 1987 and the Outpatient Register was started in 2001 [18].

To assess the frequency of amyloidosis hospitalizations, amyloidosis patients were identified from years 1997 through 2000 and they were scored for the number of hospitalizations. Among 51 patients diagnosed with secondary systemic amyloidosis, 33 (65%) were hospitalized once, 9 twice and 9 three or more times. Among 27 patients diagnosed with organ-limited amyloidosis, 14 (52%) were hospitalized once, 2 twice and 11 three or more times. Among 39 patients diagnosed with other amyloidosis, 19 (49%) were hospitalized once, 4 twice and 16 three or more times. Among 125 patients diagnosed with unspecified amyloidosis, 50 (40%) were hospitalized once, 28 twice and 47 three or more times. This population of 242 patients, first diagnosed between years 1997 and 2000, was also analyzed in terms of change of amyloidosis diagnostic subtype between the first and the last discharge diagnoses. A change was noted for 23 patients (9.5%); 15 of these were initially diagnosed with unspecified amyloidosis, which was changed to ‘other’ amyloidosis among 7 patients and to hereditary amyloidosis among 4 patients (1.6% of 242 patients).

**Figure 2:**
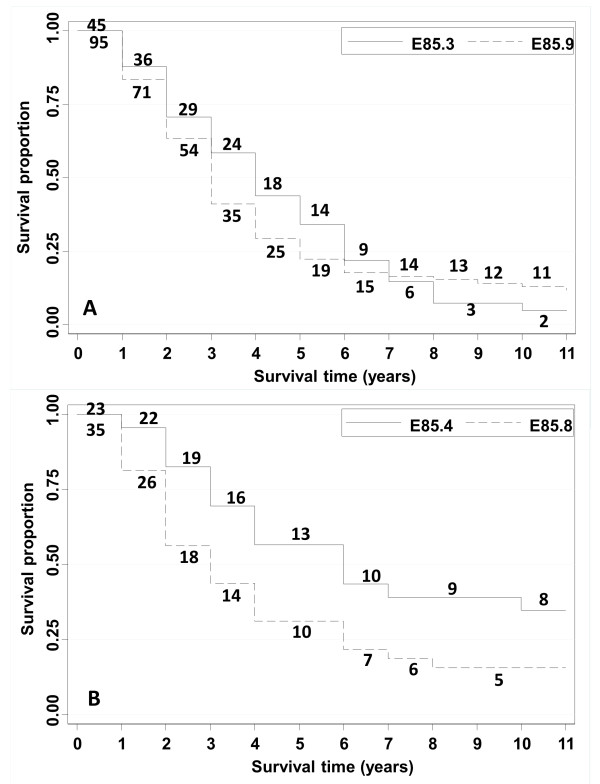
**Kaplan-Meier survival estimates for the subtypes of hospitalized patients with amyloidosis by subtypes.** The figures next to the graphs show the patients at risk in each year of follow-up. Figure 2 top: Secondary systemic amyloidosis (E85.3) and amyloidosis, unspecified (E85.9); Figure 2 bottom: Organ-limited amyloidosis (E85.4) and other amyloidosis (E85.8).

Kaplan-Meier survival curves were plotted for patients first diagnosed between years 1997 to 2000 according to their last discharge diagnosis from 1997 to 2008 (Figure 2). Figure 2A shows the curves for secondary systemic amyloidosis (median survival time 4 years) and unspecified amyloidosis (median survival time 3 years). Figure 2B shows the curves for organ-limited amyloidosis (median survival time 6 years) and ‘other’ amyloidosis (median survival time 3 years).

## Discussion

The present study helps to fill in the gap of knowledge regarding the incidence of amyloidosis [17]. The advantages include nation-wide coverage in a country of universal access to medical services of high diagnostic standards. Many patients were discharged more than once with the main diagnosis amyloidosis, which implies consistent diagnostics. Among those whose underlying cause of death was amyloidosis, 77% had been earlier discharged with a diagnosis of amyloidosis, which should provide a minimal estimate on the degree of hospitalization of these patients in Sweden. However, considering the seriousness of amyloidosis and the related diagnostic challenges, it is likely that almost all AL amyloidosis patients would be hospitalized. The accuracy of death certificates is also high in Sweden because many deaths take place in hospitals and hospital records are available to the death registrar in some 80% of the fatalities in diseases covering amyloidosis [19].

Diagnostics of amyloidosis is difficult and time consuming, relying on tissue biopsy and microscopic examination [3]. Fine needle aspiration biopsy from a symptom-giving organ can be used but subcutaneous adipose tissue offers a safe and more convenient alternative which with Congo red staining has a diagnostic sensitivity of 70 to 90% [3]. The diagnostic verification of AL amyloidosis should involve multiple steps: immunofixation electrophoresis in combination with serum free light chain assay to identify the light chain, followed by bone marrow examination to exclude multiple myeloma and Congo red staining of bone marrow or subcutaneous fat to identify amyloid deposits; these procedures should verify some 90% of patients [4]. It is understandable that the diagnosis may change after the first hospital visit and, for that reason, we used the last available discharge diagnosis. However, the changes appeared to be few, probably because the amyloidosis subtypes categorized by the ICD-10 codes were anyway non-specific and not informative of the nature of the amyloid fiber. The final diagnosis of hereditary amyloidosis was found in only 1.6% of patients with the initial diagnosis of unspecified amyloidosis. Hereditary amyloidosis FAP is well known in Sweden because the country is one of the high prevalence areas for FAP globally [12,13]. Reassuringly, the present patient series showed no excess incidence in the two northernmost provinces which are the endemic area of FAP [13]. Data from the UK have shown that hereditary amyloidosis may be misdiagnosed as AL amyloidosis [20].

Nevertheless, we acknowledge that the available disease classification in ICD-10 is not unambiguous. Based on the observation that 10% of AL amyloidosis patients have a concomitant myeloma [16], we calculated in Results that AL amyloidosis would account for 38% of all cases and correspond to an incidence of 3.2 per million person-years. It was similarly assumed that AL amyloidosis constituted some 80% of ‘other’ amyloidosis, which showed a median survival time of 3 years. The median survival for AL amyloidosis in an Italian clinic was reported at 3.8 years but even shorter survival times have been recorded, depending on the staging and course of the disease [2,4,6]. The relatively favorable survival in the present study can also be explained by the recent follow-up (after 2001) and the improvements of treatment. Amyloidosis and myeloma treatments are principally similar, and increases in myeloma survival have been ascribed to the treatment [3,21].

Rheumatoid arthritis could be the main cause of secondary systemic amyloidosis. Indeed, the largest number of rheumatoid arthritis deaths (14) occurred in secondary systemic amyloidosis but an additional 10 deaths occurred in unspecified amyloidosis. Further support for a large rheumatoid arthritis etiology in secondary systemic amyloidosis was the female excess and a relative good survival. Thus, of the total amyloidosis incidence of 8.29 per million person-years, close to 1 per million could be assigned to secondary systemic amyloidosis with a median survival of 4 years. However, an almost equal number of rheumatoid arthritis cases would be contributed by unspecified amyloidosis and the best estimate for secondary amyloidosis incidence would be 2 per million person-years. It is however clear that rheumatoid arthritis related secondary amyloidosis is underdiagnosed or underreported because there are some 50,000 hospitalized rheumatoid arthritis patients in Sweden since 1964 [22]. Accordingly, amyloidosis incidence in rheumatoid arthritis patients in Finland has been 18 per million person-years when systematic amyloid determination was conducted [11]. The median survival time was 6 years in that study and it had been 3 years in an earlier Finnish study which probably shows that the control of the inflammation has a positive effect on survival [10,23]. This was confirmed in a recent study from Finland [24].

Senile amyloidosis is characterized by male excess and age of onset higher than 65 years [4]. Practically all subtypes in the present study were characterized by a high age male incidence peak and it is not possible to assign any incidence estimate to senile amyloidosis. Organ-limited amyloidosis may be caused by many types of amyloid deposits and it could not be assigned to a specific amyloidosis subtype [25]. The 6 year median survival for this group is commensurate with limited disease severity.

## Conclusions

This nation-wide study showed an incidence of 8.29 per million person-years for non-hereditary amyloidosis in Sweden. This is close to the incidence of AL amyloidosis (9 per million) reported for the Mayo Clinic in the USA [17]. Although the present diagnostic categorization was too crude to single out AL amyloidosis in the Swedish discharge data, we could extrapolate from myeloma rates that the incidence of AL amyloidosis was 3.2 per million person-years. The other disease entity that could be discerned was rheumatoid arthritis related secondary systemic amyloidosis with an estimated incidence of 2 per million. The unaccounted incidence of about 3 per million person-years may be accounted for by senile amyloidosis of the error margins of the above two amyloidosis subtypes. We have reasons to believe that these incidence figures were minimally influenced by hereditary amyloidosis. Median survival ranged from 3 to 6 years and it was worse for the subtype with most assumed AL cases. Amyloidosis epidemiology will be boosted when the new amyloid fibril based disease nomenclature will be taken to clinical use and supported by diagnostic know-how [1].

## Competing interests

The authors declare that they have no competing interests.

## Authors’ contributions

KH, XJ and KS designed the study, JS, KS, XJ and KH gathered and analyzed the data, KH, AF and XJ wrote the report and all authors comments on the manuscript. KH confirms that he had full access to all the data in the study and had final responsibility for the decision to submit for publication. All authors agreed to publishing the paper. All authors read and approved the final manuscript.

## Pre-publication history

The pre-publication history for this paper can be accessed here:

http://www.biomedcentral.com/1471-2458/12/974/prepub
